# Resveratrol natural product inspired compound as a potent neuroprotectant against acute oxidative stress

**DOI:** 10.17912/micropub.biology.001127

**Published:** 2024-09-03

**Authors:** Alec Simonson, Akshay Naraine, Samantha Maki, Kristina Nugent, Salvatore Lepore, Ken Dawson-Scully

**Affiliations:** 1 Department of Biological Sciences, Florida Atlantic University, Boca Raton, Florida, United States; 2 Max Planck Florida Institute for Neuroscience, Jupiter, Florida, United States; 3 Department of Chemistry and Biochemistry, Florida Atlantic University, Boca Raton, Florida, United States; 4 Department of Psychology and Neuroscience, Nova Southeastern University, Fort Lauderdale, Florida, United States

## Abstract

Resveratrol as well as natural products biosynthetically derived from it, have been shown to have protective effects against oxidative stress. However, these compounds possess poor druglike properties. At sub-nanomolar concentrations, a novel compound (RVM-6) inspired by a resveratrol natural product prolongs synaptic viability in neuromuscular junctions in *D. melanogaster* exposed to acute oxidative stress.

**Figure 1. 1a) Structure of RVM-6. 1b) RVM-6 improves synaptic viability in larvae exposed to oxidative stress.**
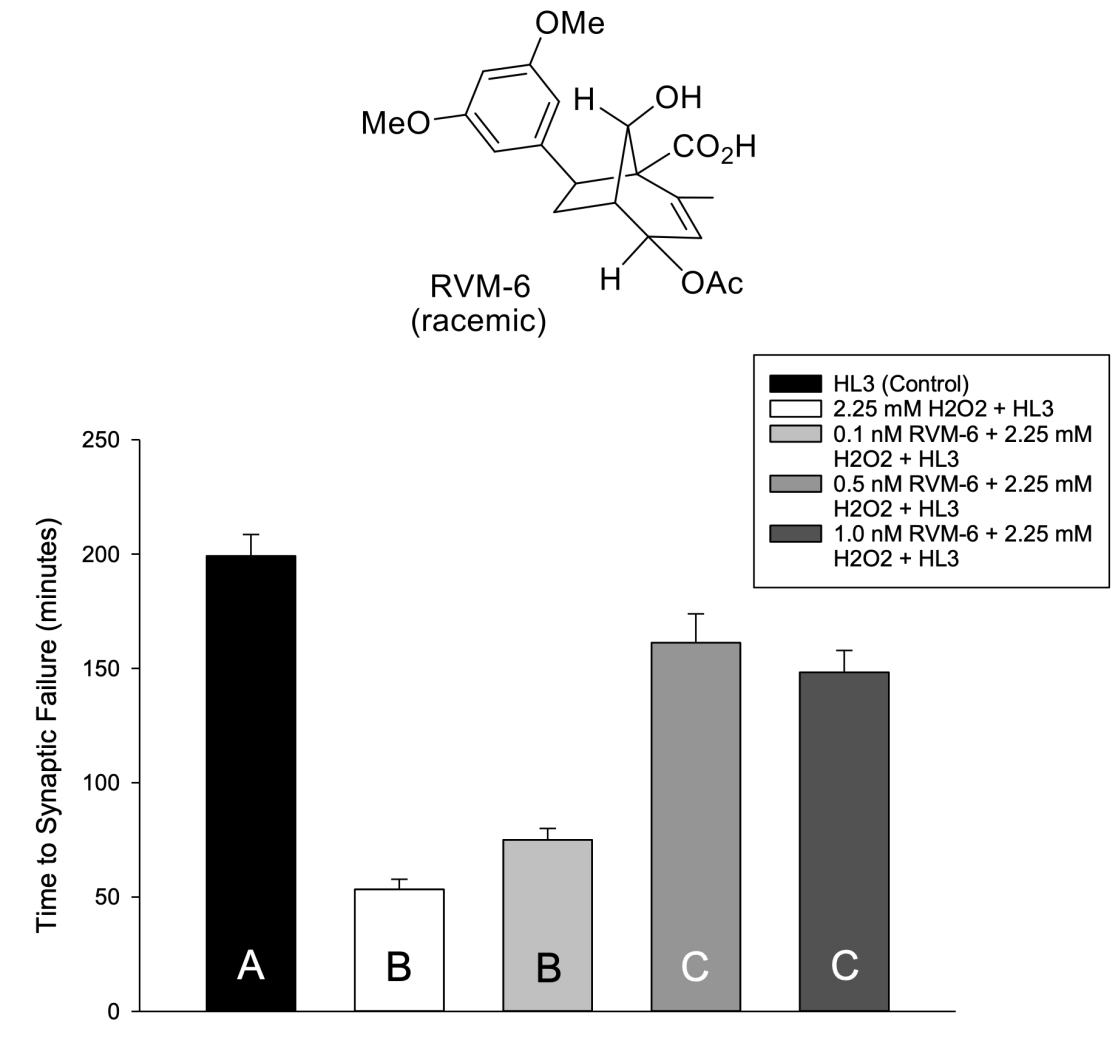
a) Structure of RVM-6 prepared in the Lepore Laboratory. b) Average times to synaptic failure are shown for w ^1118^ *D. melanogaster* 3 ^rd^ -instar larvae at different concentrations of Resveramorph 6 (RVM-6) in solution (total HL-3 n = 6; 2.25 mM H _2_ O _2_ n = 6; 0.1 nM RVM-6 + H _2_ O _2_ n = 2; 0.5 nM RVM-6 + H _2_ O _2_ n = 4; 1.0 nM RVM-6 + H _2_ O _2_ n = 6). HL-3 saline was a sham condition and positive control, and 2.25 mM H _2_ O _2_ was used as a negative control. Time to synaptic failure is the time in which it takes the excitatory junction potential to fall below a 1.00 mV threshold. Larvae exposed to the HL-3 sham condition averaged 199.2 minutes to reach synaptic failure. Animals exposed to 2.25 mM H _2_ O _2_ averaged 53.3 minutes to synaptic failure. Larvae exposed to 0.1 nM RVM-6 + H _2_ O _2_ averaged 75 minutes to reach synaptic failure. Larvae exposed to 0.5 nM RVM-6 + H _2_ O _2_ averaged 161.3 minutes to reach synaptic failure. Animals exposed to 1.0 nM RVM-6 + H _2_ O _2_ averaged 148.3 minutes to synaptic failure. Data are means ± the standard error of the mean (SEM). Statistical significance (p < 0.05) was designated utilizing letters where different letters indicate statistical differences, and the same letter indicates non-significance; one-way ANOVA and Holm-Sidak posthoc analysis.

## Description

*Drosophila melanogaster* has been an effective model to study disease and oxidative stress tolerance (Armstrong et al., 2011; Mirzoyan et al., 2019; Ugur et al., 2016) . Of particular interest, the neuromuscular junction in *Drosophila melanogaster* utilizes glutamate as an excitatory neurotransmitter, which likens it to the human central nervous system (Ataman et al., 2006; Menon et al., 2013; Ugur et al., 2016) . One of Drosophila’s major benefits as an invertebrate model is for novel drug screening; both third instar larvae and adult flies have been well described as powerful drug discovery tools, especially for neuroprotective compounds (Millet-Boureima et al., 2021; Newman et al., 2011; Pandey & Nichols, 2011) .

Resveratrol natural products are well known for their antioxidant activities (Matsuura et al., 2015) . For example, the resveratrol dimer ε-viniferin is thought to function as a radical scavenger and its isoprenylated analogs have been demonstrated to exhibit inhibitory activity against human monoamine oxidase B (hMAO-B) leading to neuroprotective effects against ROS generation and hydrogen peroxide-induced apoptosis, albeit at micromolar-level potencies (Shang et al., 2022; Tang et al., 2019) . Nevertheless, the polyphenolic structures of resveratrol natural products often lead to rapid hepatic clearance, as was demonstrated in the case of ε-viniferin, making them poor candidates for further development as drugs (Courtois et al., 2017) . As part of a program to create more druglike compounds inspired by resveratrol natural products, we previously disclosed two novel [3.2.1] bicyclic compounds (Bollinger et al., 2019) . In this work, using drosophila larva electrophysiology recordings, we have now examined RVM-6 to further explore this compound class ( Fig. 1 a). We found that the effective threshold for RVM-6 against an oxidative stressor was 0.5 nM, and we did not detect any deleterious effects at twice that dosage ( Fig. 1 b).

Though the mechanism of RVM-6 remains an area of investigation, this compound exhibits protective effects in the sub-nanomolar range. The only other reported neuroprotective small molecule (AND-302) showed no activity at 0.5 nM, well above its reported effective dose (0.09 nM) in hippocampal cell cultures (Smith et al., 2014) . The current results offer encouraging evidence that the RVM compound system is worthy of further study as neuroprotective agents. Though some groups in the dataset are limited by sample size, the current pilot study’s findings show little variance and are in line with previously published data (Bollinger et al., 2019) . These findings serve to provide informative preliminary data that will spur future studies and inquisition about structure-activity relationships for future RVM compounds. The next step will be to examine more RVM analogs and perform a comprehensive SAR analysis to elucidate the pharmacokinetic properties of this family of compounds.

## Methods

*Drosophila* larva electrophysiology: Individual *Drosophila* larvae were collected and placed on a glass-dissecting plate containing ~2 mL of Schneider's insect medium (Sigma, St. Louis, MO). Each larva was positioned with the dorsal side up on the dissecting dish using standard insect pins. Removal of the internal organs and central nervous system was achieved by making a longitudinal cut in the anteroposterior direction along the dorsal surface to expose the underlying segmental muscles and nerves. An extracellular glass suction electrode was used to stimulate segmental nerves in muscle segments. The postsynaptic excitatory junction potential (EJP) was recorded from muscle 6 with a sharp intracellular glass recording electrode filled with 3 M KAc (∼40 MΩ).

The preparation medium was replaced with HL-3 saline (1.5 mM CaCl _2_ , 20 mM MgCl _2_ , 5 mM KCl, 70 mM NaCl, 10 mM NaHCO _3_ , 5 mM BES, 115 mM sucrose, 5 mM trehalose·2H _2_ O) made fresh daily (Macleod et al., 2002; Stewart et al., 1996) . EJP recordings were viewed with an oscilloscope and digitally stored using the Scope program (AD Instruments, Colorado Springs, CO) for analysis. Evoked EJPs from repetitive stimulation (0.3-ms pulses delivered suprathreshold with a 1-Hz frequency) of both axons in larval muscle 6 were recorded in a stop-flow condition. EJP recordings were taken until synaptic transmission failure (amplitude <1 mV) occurred.

Intracellular recordings of the resting membrane potential (RMP) and input resistance were measured from larval muscle 6 with signals amplified by an IX1 intracellular preamplifier (Dagan, Minneapolis, MN). These measurements were taken as previously described (Zhang & Stewart, 2010) . Briefly, RMP measurements were taken from animals if the initial potential stabilized between −60 to −70 mV. If the membrane potential was equal to −45 mV or more depolarized, the preparation was discarded. Input resistance was measured by injecting small current pulses of 2 nA (40-ms duration at 1-Hz frequency) applied continuously. Electrode resistance was canceled prior to measuring the input resistance by adjusting the bridge balance control. Resistance was calculated using Ohm's law and only muscle fibers with an initial input resistance >5 or <40 MΩ were assayed.

Pharmacological manipulations: Drugs were dissolved directly into HL-3 saline solution and about 5 mL of solution were aliquoted into a transparent tube. Pharmacological manipulations were completed using a bath switch from HL-3 to HL-3 with drug manipulations at 4-5 minutes into trial after recording saline baseline data. HL-3 with drug manipulation remained a continuous exposure throughout the remainder of the trial. The compounds tested was RVM-6. RVM-6 is a novel compound; however, its synthesis followed a previously published procedure (Bollinger et al., 2019) . Additionally, following a previous disclosure (Smith et al. 2014) , AND-302 was synthesized.

## Reagents

Strain: w ^1118^ (3 ^rd^ instar larvae only)

Chemicals: H _2_ O _2_ (CAS: 7722-84-1), RVM-6 and AND-302 (synthesized and provided by the Lepore Group)
